# Lasers and intense pulsed light (IPL) association with cancerous lesions

**DOI:** 10.1007/s10103-017-2310-y

**Published:** 2017-09-07

**Authors:** Caerwyn Ash, Godfrey Town, Rebecca Whittall, Louise Tooze, Jaymie Phillips

**Affiliations:** University of Wales Trinity Saint David, Swansea, SA1 6ED UK

**Keywords:** Intense pulsed light (IPL), Actinic keratosis (AK), Ultraviolet (UV), Basal cell carcinoma (BCC), Malignant melanoma (MM)., Squamous cell carcinoma (SCC)

## Abstract

The development and use of light and lasers for medical and cosmetic procedures has increased exponentially over the past decade. This review article focuses on the incidence of reported cases of skin cancer post laser or IPL treatment. The existing evidence base of over 25 years of laser and IPL use to date has not raised any concerns regarding its long-term safety with only a few anecdotal cases of melanoma post treatment over two decades of use; therefore, there is no evidence to suggest that there is a credible cancer risk. Although laser and IPL technology has not been known to cause skin cancer, this does not mean that laser and IPL therapies are without long-term risks. Light therapies and lasers to treat existing lesions and CO_2_ laser resurfacing can be a preventative measure against BCC and SCC tumour formation by removing photo-damaged keratinocytes and encouraged re-epithelisation from stem cells located deeper in the epidermis. A review of the relevant literature has been performed to address the issue of long-term IPL safety, focussing on DNA damage, oxidative stress induction and the impact of adverse events.

## Introduction

The development and use of lasers and light for medical and cosmetic procedures based on the principle of selective photothermolysis [1] has increased exponentially over the past two decades. Since the first commercial cutaneous carbon dioxide laser system became globally available over 40 years ago [2], millions of treatments have taken place often with positive outcomes. This review will look into the research on how light therapies affect cancerous and precancerous skin lesions with the aim of determining whether such treatments could ever initiate cancer or make an existing cancer lesion worse. The notable increase in the cosmetic and medical uses of lasers and IPL systems are not restricted to physicians, as many home-use devices are available with comparable parameters to salon-based equipment [3, 4].

Following the first demonstration of a functional laser device in 1960, it was not until the early 1990s that their use in the medical field became commonplace. Although laser interaction with skin and tissues was investigated from the mid-1960s onwards, the high cost and unreliability of the technology restricted its commercial uptake [5].

In the late 1980s, lasers that targeted specific constituents of tissue, rather than just cutting or ablating, were introduced for the removal of vascular lesions [6, 7]. Throughout the early 1990s, various lasers were introduced for different applications in the dermatology field, ranging from tattoo removal to hair removal. The IPL system is a high power broadband light source as opposed to the monochromatic laser, made its debut in the mid-1990s as an alternative to the available laser sources. The perceived advantages of the IPL were lower cost, less complexity and more flexibility in output parameters along with the incoherent nature of the IPL source compared to the focusing effect on the retina by coherent lasers being generally less hazardous to the eye.

Hair removal using monochromatic lasers was initially introduced to the market in the early 1990s [8, 9]. However, in the new millennium, broadband IPL technology emerged as a powerful competitor and challenged the dominance of the laser in the market [10]. A notable reason for the increasing popularity of IPLs amongst dermatologists and beauty therapists is versatility. An IPL system can be configured for different emission spectra by simply varying light filtration. A distinct advantage is the ability to target multiple chromophores over a large skin area in a single pulse compared with narrow beam, small spot size laser systems. Initially, IPLs were utilised for the treatment of vascular malformations such as port wine stains and thread veins. This was because they had the ability to produce longer pulse durations than pulsed dye lasers, the technology most commonly used for vascular treatments, thus allowing larger veins to be targeted and treated. As the 1990s progressed, the use of IPLs became more and more commonplace with photodermatological treatments, with the exception of tattoo removal, being addressed by a wide variety of systems with a myriad of output parameters.

IPL-based technology is generally considered a safe procedure as potentially harmful ultraviolet radiation is typically filtered by blocking wavelengths below 500 nm, although there are unwanted effects associated with the treatment. Adverse effects of IPL treatments have been well documented [11]; these include blistering, hypopigmentation, hyperpigmentation and if extensive, even scarring. Hyperpigmentation is typically reversible and results from the stimulation of melanin from epidermal melanocytes and is similar to that seen in an ultraviolet light-induced suntan. Hypopigmentation may be permanent and it usually results from thermally induced destruction of melanocytes. A majority of cases occur due to incorrect parameters being selected for the treatment based on the application and patient’s skin colour or ethnicity [12]. Laser and IPL systems all utilise non-ionising radiation; this type of energy does not affect the DNA strands. It is this breakdown of DNA which may lead, in the long term, to cancer. The evidence base suggests that direct DNA damage is restricted to ultraviolet (UV) wavelengths < 400 nm, which is much shorter than the wavelengths commonly emitted by IPL devices [13].

Since the introduction of IPL devices, wavelengths below 400 nm have, in the vast majority of cases, been filtered out. Xenon arc flashlamps, the primary lamp type used in IPLs, do produce wavelengths shorter than 400 nm but through the use of doped flashlamp glass envelopes, wavelengths below 360–380 nm are prevented from exiting the flashlamp wall. Additional filters are used in the optical path to remove wavelengths from 380 nm to the desired cut-off wavelength, usually between 500 and 600 nm depending on the system and application. Wavelengths between 400 and 500 nm have been utilised in IPL devices, most commonly for the treatment of acne and psoriasis, that target the absorption band of porphyrin in the 400–420-nm region. Twenty-five years ago, mothers were concerned that the first laser treatments for port wine stain birthmarks would cause cancer later in life; this has now been dismissed.

As the wavelengths utilised by the majority of commercially available IPLs have been generally above 500 nm, combined with the well-documented DNA damage action spectrum being below 400 nm, there has been very little research undertaken on the impact, if any, of IPL devices on direct DNA interactions.

Since 1995, the PubMed database contains references for close to 500 peer-reviewed publications detailing experiences with IPL devices in the professional arena. Since the first publication of IPL use on skin in 1995, the evidence base consists of over 20 years’ worth of publications and studies were performed with a wide variety of IPL systems and parameters and, to date, there has been no indication that repeated exposure or cumulative adverse events may lead to potential long-term risks. Therefore, a review of the relevant literature has been performed specifically to address the issue of long-term IPL safety, focussing on DNA damage, oxidative stress induction and the impact of adverse events.

## Review

There are three major types of skin cancer: basal cell carcinoma (BCC), squamous cell carcinoma (SCC) and malignant melanoma (MM). Since these are the most commonly observed, they are also the most studied in terms of their reaction to light therapies. Basal cell carcinoma is a cancer of the cells at the deepest part of the epidermis. BCCs account for 75% of all skin cancers in the UK; they are slow to grow and rarely metastasize. BCCs can be difficult to diagnose due to their position deep in the epidermis as the top layer can remain intact whilst the BCC grows underneath. However, BCCs, if caught early, are very treatable and most patients are completely cured after treatment. Squamous cell carcinoma is a cancer of the outermost cells of the epidermis. SCCs account for 20% of skin cancers in the UK, and they are also slow growing and again rarely metastasize unless left untreated for an extended period of time [14]. Sometimes the appearance of a SCC is preceded by a lesion called an actinic keratosis (AK). These AKs are small-coloured rough spots usually occurring in the skin that has been frequently exposed to the sun. Around 5 to 10% of AKs go on to become skin cancer (SCC type); however, it is currently not possible to determine which ones will develop into a cancer but AKs never turn into malignant melanomas [15]. Due to the risk of AKs becoming cancerous, various treatment options are considered in removing the affected cells including topical agents. Surgery and cryosurgery are commonly used options; however, photodynamic therapy (PDT) using light-emitting diode (LED) arrays and IPL systems has recently become preferred choices for patients because of their efficacy and lack of serious complications that can come with invasive surgery.

Using IPL for PDT itself will damage the tissue; however, this PDT damage is mainly restricted to the cells and not to the connective tissue, so that the healing process is significantly improved as the connective tissue is not damaged.

A study by Avram and Goldman in 2004 found that combining IPL with a photosensitiser (ALA) meant 68% of the treated AKs resolved after only one treatment [16]. Furthermore, a study performed by Kim et al. in 2005 also used IPL with ALA and histologically confirmed that 42% of the treated AKs were cleared in one treatment. They concluded that this clearance rate would improve if further treatments were performed but warned that longer-term studies would be needed to confirm complete remission [17]. In addition to using light therapies and lasers to treat existing lesions, research has been conducted into using laser resurfacing as a preventative measure against BCC and SCC tumour formation. Massey and Eliezri executed a case study involving two patients; one had multiple facial BCCs and the other numerous SCCs on the face and scalp. They administered CO_2_ laser resurfacing on both patients, completing two passes on all areas of the face except the eyelids and nasal bridge which received one pass. They found no new skin cancers and only one AK within the treated area 33 months (patient 1) and 52 months (patient 2) after the laser resurfacing treatment. Interestingly, the authors say both patients developed new tumours in areas not treated in the study. They concluded that CO_2_ laser resurfacing effectively removed photo-damaged keratinocytes and encouraged re-epithelisation from stem cells located deeper in the epidermis. These cells may have been protected from sun damage by the more superficial keratinocytes. Forcing epidermal regeneration with cells that have normal cell differentiation prevents the formation of new tumours, therefore proposing that laser resurfacing could be a suitable treatment option in the prevention of skin cancer formation in certain patients [18].

MM is a cancer of the cells that produce melanin or melanocytes. Melanoma will present as a spreading dark spot or mole on the skin and is caused by the melanocytes growing out of control. Unlike BCCs or SCCs, melanoma is classed as a malignant condition or faster growing and prone to metastasize. MM is responsible for 75% of the deaths associated with skin cancer, emphasising that it is the most dangerous of the skin cancer types [19].

Despite the many studies that conclude the opposite, there is still a concern about the formation of skin cancer using light therapies. Hedelund et al. performed a study on mice which tried to induce carcinogenesis with IPL treatment [20]. They also looked at whether UV exposure influenced the IPL side effects. They exposed the animals to simulated solar radiation, IPL treatments or both. They also had a control group that remained untreated. They found that skin tumours developed in the groups exposed to the UV radiation regardless of whether they had also been exposed to the IPL treatment. No tumours were found in the control group or the group only treated with IPL. Their study advocates that IPL treatment alone has no carcinogenic potential as the wavelengths present in IPL systems are outside of the carcinogenic spectrum in the UV range. They did find that pre-treatment UV exposure increased the incidence of oedema and erythema following the IPL treatment, confirming the already well-established advice that patients restrict UV exposure before and after IPL treatment [20]. Moreover, Werneck et al. conducted an in vitro study with cancer cells to investigate whether laser light could induce proliferation. They established that wavelength had the greatest influence on cancer cell growth and that time of irradiation only mattered at the shortest wavelength studied. This reinforces that with respect to cancer induction, the dominant factor is wavelength of the light of the skin is being exposed to [21].

All of the studies mentioned so far have concentrated on non-melanoma skin cancer. Pinheiro et al. conducted a study looking at the effect of low-level laser therapy on malignant cells in vitro. In particular, they studied epithelial-like cancer cells exposing them to diode laser light with wavelengths of 635 and 670 nm. They found that the irradiated cells (both wavelengths) proliferated more than the non-irradiated control group. The 670 nm exposed group did proliferate more than the 635 nm group. The authors therefore concluded that laser light exposure could significantly increase proliferation of cancer cells [22]. In vivo studies looking into malignant melanoma are not easily accomplished; however, Gottschaller et al. presented the accidental exposure of a malignant melanoma with a CO_2_ laser as a case study. The patient was misdiagnosed as a lentigo simplex and the lesion was treated by CO_2_ laser vaporisation. Three years later, the patient presented with a metastasis of a malignant melanoma with no evidence of the primary melanoma. The authors deduced that is it unclear whether the initial lesion was a malignant melanoma or whether a malignant melanoma was induced by the laser treatment. They therefore advise that any suspicious lesions should be removed surgically and histologically examined, as laser-induced progression to malignant melanoma cannot be ruled out [23]. Zipser et al. [24] discussed the outcome of 12 patients presenting with melanoma with previous use of laser treatment. Five patients at the University Hospital Zurich and 7 from a literature review were used to calculate a mean time between treatment and diagnosis from a range of 18 to 144 months. However, with a limited sampling and uncertainty, whether the laser treatment was a contributor in the development is unknown.

It appears that the only relevant study on DNA damage by IPL to date was undertaken by Sorg et al. (2007). Specifically, Sorg et al. looked at the effect of IPL irradiation on the production of Thymine Dimers molecular lesions formed from thymine or cytosine bases in DNA—indicative of photon-induced damage. Exposing nine subjects to IPL light with a wavelength range of 520–750 nm, 9 J/cm^2^ fluence and pulse duration of 2.5 msec, Sorg et al. showed, through histological evaluation, no evidence of Thymine Dimer production when compared to control or UV-A-exposed sites [25]. Sorg et al. determined that high-peak intensity visible light applied to the skin, with the wavelength, pulse duration and fluence parameters described, did not result in Thymine Dimer production, a key marker of DNA damage whose formation has a direct link to an elevated risk of skin cancers. The findings of Sorg et al. are supported by Chan et al. [26], who looked at repeated skin exposure to the various light and laser sources commonly used in non-ablative skin rejuvenation (skin rejuvenation without disruption to the skin surface). The three light sources used were the pulsed dye laser emitting 585 nm yellow light, Nd:YAG infrared laser at 1320 nm and an IPL with a low-end cut-off filter at 500 nm. The energy delivered to the skin varied from 8 J/cm^2^ (dye laser) to 30 J/cm^2^ (IPL) with pulse durations from 1.5 msec (dye laser) to 20 msec (IPL). All treatments were performed on a twice-weekly basis for 6 months, a total of 52 exposures. Histological analysis of the irradiated tissue at 6 months showed no toxicity or tumour formation. Elevated levels of histological markers that are predictors of DNA damage were recorded but did not appear to lead to other abnormalities. The increase in p16 expression post treatment indicated by Chan will have no impact on the likelihood of tumour formation; elevated cancer risks are only associated with a depletion or mutation in the p16 gene, neither of which were identified in this study. In actuality, the p16 gene naturally increases with age as a cancer protection mechanism.

## Reactive oxygen species

In terms of oxidative stress, Sorg et al. also looked at the impact of IPL exposure on the production of lipid peroxides in the skin. Lipid peroxides result from the oxidative degradation of lipids found in cellular membranes. Reactive oxygen species (ROS), which are short-lived highly reactive species, including OH, HO_2_, O_2_- and O_2_ (1D), interact by oxidising their surroundings. O_2_ in its singlet delta lowest electronically excited state, O_2_ (1D), may for instance insert into a C–H bond to form COOH. Thus, basically, these reactive oxygen species interact with their surroundings by oxidising them. This may for instance happen by reaction with the molecules in a lipid membrane of a cell resulting in a lipid peroxide, and may lead to significant cell damage [27].

The body’s natural antioxidants, such as vitamin E, can control the level of cellular injury by effectively “catching” any free radicals, thereby protecting the body from further damage.

Sorg et al. demonstrated that at the high peak powers used in their study, elevated lipid peroxide levels were induced following IPL irradiance when compared to both control and UV-A exposure. As the output wavelength spectrum contained no UV and very little infrared (IR) light, it was determined that lipid peroxides were indeed induced by the visible portion of the spectrum.

The data from Sorg et al. revealed that the IPL induced a higher formation of lipid peroxide than UV-A exposure (315–400 nm), with the IPL resulting in approximately twice the level of that of UV-A light at these IPL parameters. Unfortunately, Sorg et al. did not undertake any dose-ranging studies for either UV-A or IPL irradiation, only relying on a single data point for each wavelength range. However, Sorg et al. findings on lipid peroxide formation, most likely induced by ROS, are supported by Zastrow et al. (2009) who showed that ROS production is stimulated across the entire spectrum from UV to IR. Whilst the Zastrow study cannot be directly correlated to the Sorg et al. data, as the exposure mechanisms were considerably different for the visible wavelength range, it does indicate that ROS production is not, and has never been, limited to UV wavelengths [28].

Zastrow et al. established that exposure to visible wavelengths did result in ROS production by irradiating tissue with direct sunlight, including UV, then removing the UV component through the use of a suitable filter. In this case, Zastrow identified that approximately 50% of the ROS production in the skin was from wavelengths greater than 430 nm, later determined that the dominant spectral band in the visible region being between 410 and 490 nm. This finding is comparable to that of Eicher et al. [29] who analysed the effects on cells when directly illuminated by broadband light. Breaking down the spectrum into three distinct bands, namely UV (< 400 nm), blue (400–500 nm) and other (> 500 nm), the data concluded that the 400–500 nm band induced “oxyradical” or ROS production. Although there is good tissue penetration of the red wavelengths at 630–635 nm which are produced by all IPL systems, the absorption of porphyrins near 630–635 nm is not as strong as in the porphyrin’s Soret band near 400 nm. With their detection method, Zastrow et al. could show no quantifiable ROS levels in their study [28]. However, significant levels of ROS will definitely be produced by IPL at 630–635 nm when porphyrins are present.

A publication by Jung et al. (2010) showed that ROS production within cultured human fibroblast cells was directly proportional to the induced temperature rise. When the cell temperature remained constant, irrespective of the light dosage applied (in this case, IR light between 780 and 1400 nm), ROS production was negligible when compared to uncontrolled temperature conditions. In addition, Jung showed that heat alone could induce significant levels of ROS [30].

If, as both Jung and Zastrow imply, the production of ROS is temperature dependant, then the peak power applied to the tissue would dictate the resultant ROS levels. The temperature rise in the tissue when exposed to light is directly dependant on wavelength, pulse duration and fluence. Therefore, it would be factual to propose that when the wavelength and fluence are similar, the pulse duration, and hence the peak power, is the overriding factor.

## Long-term risks

A full-thickness or third degree burn would be the most severe adverse event that could occur to the skin through misuse, error or malfunction of an IPL or laser. It has been acknowledged over many years that the incidence of skin cancer is elevated in burn victims and Marjolin’s ulcers [31], the most commonly cited reference being the 1930 publication by Treves and Pack [32]. Two recent large-cohort studies have dispelled this belief, both based upon more reliable data of direct patient records. Mellemkjaer et al. (2006) reported a review of 16,903 burns victims whose injuries occurred during the period 1978 to 1993. With up to 25 years patient follow-up (mean of 15.6 years), the incidence of SCC, MM or any other form of skin cancer was not outside the expected norms [33].

This study was supported by further work by Lindelof et al. (2008) in a population-based cohort of 37,095 subjects hospitalised for burns over the period 1964 to 1996. With a mean follow-up period of 16.4 years (range 0 to > 39 years with over 12,700, or 34% of subjects, followed for > 20 years), Lindelof et al. reported a Standardised Incidence Ratio (SIR) for SCC of 0.88 (95% CI 0.70–1.09) and for MM the SIR was also 0.88 (95% CI 0.68–1.12), both below the expected population incidence norm [34].

Mellemkjaer and Lindelof together concluded that burn injuries did not increase the likelihood of developing malignant forms of skin cancer. Although it could be argued that the Mellemkjaer study follow-up period was less than the average time to cancer formation quoted in the Kowal-Vern review, the Lindelof study was of sufficient duration and power to highlight any causal relationship between the burn and tumour formation.

## Discussion

The use of photodynamic therapy and IPL systems in the treatment of AKs, BCCs and SCCs is supported by studies concluding that their application is safe and effective. In the case of MM, the conclusions are not so apparent. In vitro studies confirmed that laser irradiation could cause an increase in cell proliferation, and therefore, treatment of MM with light could worsen the condition. However, there is a lack of in vivo data for comparison. The majority of the data gathered has been suspected accidental exposure of a primary MM but as the initial lesion may have been destroyed by the original treatment, this cannot be proven. What is evident is that the patients present with malignant melanoma after an initial treatment. Whether this original lesion was a malignant melanoma or something more benign is still a matter of debate. A report by ARPANSA radiation health committee states the diagnosis of skin cancers can be missed or delayed because a pigmented lesion was incorrectly treated with IPL or laser [35]. Therefore, definitive conclusions about whether the treatment caused the cancer or that the cancer was present previously and the treatment had no impact, cannot be drawn. This lack of evidence and the difficulty in obtaining more relevant in vivo data suggests that light-based treatment of malignant melanoma should be avoided and a more conventional treatment method should be used.

In addition to using light therapies and lasers to treat existing lesions, CO_2_ laser resurfacing can be a preventative measure against BCC and SCC tumour formation by removing photo-damaged keratinocytes and encouraged re-epithelisation from stem cells located deeper in the epidermis.

The relative radiant exposure and wavelengths of IPL systems are outside the parameters of the widely accepted photochemical mode of action of inducing carcinogenic effects [3, 16]. IPL systems do not cause significant dermal damage compared to ablative lasers as they have limited power. It has been postulated that ablative lasers may conceal lesions in the dermal layer delaying treatment [36]. Given the aggressive nature of malignant melanoma, the number of skin rejuvenation treatments worldwide and the low number of anecdotal cases with a mean delay of 36.1 months [20], it seems unlikely a link between treatment and formation of MM as a direct result of laser or IPL treatments.

## Conclusions

Typical IPL device settings will not have a photochemical or photothermal reaction with melanomas. The existing evidence base of over 25 years of laser and IPL use to date has not raised any concerns regarding its long-term safety with only a few anecdotal cases of melanoma post treatment over two decades of use. Repeated exposures to high-intensity IPL light, the example quoted being 52 treatments over 6 months, did not result in increased carcinogenicity or tumour formation.

An adverse event, such as a dermal burn, does not increase the long-term likelihood of tumour formation at the injury site. Without any hypothetical *modus operandi* from the heating or absorption from the laser, a scientific link cannot be attributed.

Upon reviewing a broad range of literature, a conclusion has been drawn that if a dermal melanoma is treated, there will be no photochemical reaction as typical IPL wavelength outputs are not capable of doing so. Melanomas will absorb the IPL wavelengths superficially converting to heat; it is possible this will kill cells in the top layer, and it will continue to develop as it would have done without IPL treatment.

A recommendation by a dermatologist prior to any IPL or laser treatment should eliminate the possibilities of MM being present.

Home-use treatments such as fractional laser treatments may ulcerate melanoma causing the patient to seek further advice and provide appropriate treatment. Generally speaking, light-based treatments may aid quicker diagnosis of melanoma due to informed consultations with trained personnel during medical clinic or salon-based treatments.
